# The DELUX study: development of lung volumes during extubation of preterm infants

**DOI:** 10.1038/s41390-021-01699-w

**Published:** 2021-08-31

**Authors:** Leonie Plastina, Vincent D. Gaertner, Andreas D. Waldmann, Janine Thomann, Dirk Bassler, Christoph M. Rüegger

**Affiliations:** 1 Newborn Research, Department of Neonatology, University Hospital and University of Zurich, Zurich, Switzerland; 2 Department of Anesthesiology and Intensive Care Medicine, Rostock University Medical Center, Rostock, Germany

## Abstract

**Objective:**

To measure changes in end-expiratory lung impedance (EELI) as a marker of functional residual capacity (FRC) during the entire extubation procedure of very preterm infants.

**Methods:**

Prospective observational study in preterm infants born at 26–32 weeks gestation being extubated to non-invasive respiratory support. Changes in EELI and cardiorespiratory parameters (heart rate, oxygen saturation) were recorded at pre-specified events during the extubation procedure compared to baseline (before first handling of the infant).

**Results:**

Overall, 2912 breaths were analysed in 12 infants. There was a global change in EELI during the extubation procedure (*p* = 0.029). EELI was lowest at the time of extubation [median (IQR) difference to baseline: −0.30 AU/kg (−0.46; −0.14), corresponding to an FRC loss of 10.2 ml/kg (4.8; 15.9), *p*_adj_ = 0.004]. The biggest EELI loss occurred during adhesive tape removal [median change (IQR): −0.18 AU/kg (−0.22; −0.07), *p*_adj_ = 0.004]. EELI changes were highly correlated with changes in the SpO_2_/FiO_2_ ratio (*r* = 0.48, *p* < 0.001). Forty per cent of FRC was re-recruited at the tenth breath after the initiation of non-invasive ventilation (*p* < 0.001).

**Conclusions:**

The extubation procedure is associated with significant changes in FRC. This study provides novel information for determining the optimal way of extubating a preterm infant.

**Impact:**

- This study is the first to examine the development of lung volumes during the entire extubation procedure including the impact of associated events.
- The extubation procedure significantly affects functional residual capacity with a loss of approximately 10 ml/kg at the time of extubation.
- Removal of adhesive tape is the major contributing factor to FRC loss during the extubation procedure.
- Functional residual capacity is regained within the first breaths after initiation of non-invasive ventilation and is further increased after turning the infant into the prone position.

## Introduction

Although non-invasive modes of ventilation are considered superior to intubation at birth,^1^ approximately 60% of preterm infants are intubated in the first days of life.^2^ Prolonged endotracheal ventilation can lead to inflammation, tissue damage and the disruption of lung development.^3–6^ To reduce these risks, clinicians aim to extubate preterm infants to non-invasive respiratory support as soon as possible. Still, approximately 40% of preterm infants develop respiratory failure after extubation and require re-intubation, which is associated with significant morbidity, including increased duration of endotracheal ventilation, airway trauma, feeding difficulties and death.^7^ To avoid such adverse outcomes, prediction of successful extubation is crucial.

A low lung volume after extubation has been identified as an important predictor of extubation failure, emphasizing the relevance of functional residual capacity (FRC) for subsequent clinical outcomes.^2,8–11^ As conventional tools for monitoring lung volumes are inappropriate for repeated measurements in the neonatal setting, the effect of individual interventions on lung volumes during the complete extubation procedure is poorly understood and has never been described so far.

Recently, electrical impedance tomography (EIT) has become available in the neonatal population and allows non-invasive and radiation-free lung volume imaging.^12^ Impedance changes are measured in a cross-sectional slice of the thorax, which is representative for the whole lung in preterm infants.^13^ The use of EIT allows continuous monitoring of changes in ventilation and perfusion,^12^ as demonstrated previously in preterm infants.^14,15^

The primary aim of this study was to describe overall changes in end-expiratory lung impedance (EELI) as a marker of FRC during the entire extubation procedure of preterm infants. Secondary aims were (1) to assess the contribution of specific events during the extubation procedure on EELI, (2) to determine whether EELI changes are associated with cardiorespiratory parameters and (3) to evaluate the development of FRC after initiation of non-invasive ventilation in a breath-by-breath analysis.

## Methods

### Population and intervention

This was a prospective observational study performed at the neonatal intensive care unit of the University Hospital Zurich (Switzerland). Data were collected as part of a six-month quality control period between August 2020 and January 2021 to improve the extubation procedure in our unit. The Regional Ethics Committee of the Canton Zurich confirmed that this study meets the requirements for quality assurance/audit projects (Req-2020-00929) and, therefore, no informed parental consent was necessary. Infants were eligible for inclusion if they were born between 26^0/7^ and 31^6/7^ weeks gestational age, had no malformation that could impede regular lung aeration, were not directed for palliative care, and had a planned elective extubation.

We did not predefine any criteria or devices, and extubation was performed at the clinician’s discretion alone. Infants were intubated nasally and the endotracheal tube was fastened to the infant’s forehead and cheeks using adhesive tape (see Supplementary Fig. S1). During endotracheal ventilation, Dräger Babylog® VN500 ventilators (Drägerwerk AG, Lübeck, Germany) were used. Non-invasive respiratory support was either ventilator-generated (Babylog® VN500) or flow-driver-generated (fabian Therapy evolution; Acutronic Medical Systems AG, Hirzel, Switzerland). A textile electrode belt with 32 electrodes was fastened at the nipple level when the infant was turned to the supine position before extubation and EIT data were recorded with the LuMon^TM^ System (SenTec AG, Landquart, Switzerland) at a frame rate of 50.86 Hz.^16^

According to local standards, infants <28 weeks were extubated to non-synchronized nasal intermittent positive pressure ventilation and infants ≥28 weeks were extubated to nasal continuous positive airway pressure (CPAP). Settings were not mandated, and clinicians could individualize care. Patients received a loading dose of 20 mg/kg caffeine citrate before extubation to increase respiratory drive. All patients were extubated in the supine position and turned prone when the nasal interface (appropriately sized binasal prongs or nasal mask) was secured in place and the infant was stable. Tracheal suctioning before extubation was performed at the clinician’s discretion.

### Data collection

Patient characteristics and the level of respiratory support before, during and after the extubation procedure were recorded. EIT data were recorded continuously, and predefined events were timestamped. Heart rate (HR) and oxygen saturation (SpO_2_) were measured using a Masimo pulse oximeter with a 2-s averaging time (Masimo Radical 7, Masimo Cooperation, Irvine CA) and recorded with the NewLifeBox^TM^ recording system (Advanced Life Diagnostics UG, Weener, Germany) with two datapoints per second. Fraction of inspired oxygen (FiO_2_) and exhaled tidal volumes (*V*_T_) before extubation were extracted from the local patient data management system (Metavision, iMDsoft®, Tel Aviv, Israel). Patients were video-recorded to accurately correlate events with EIT data.

### Data analysis

Clinical, physiological and EIT data were extracted over a timeframe of 30 s for the following predefined events: immediately before first handling of the infant (*baseline*), tracheal suctioning (*suction*), start and end of adhesive tape removal (*adhesive tape begin* and *adhesive tape end*), pulling the endotracheal tube (*extubation*), initiation of non-invasive ventilation (*NIV*), immediately before and after turning the infant to prone position (*supine* and *prone*, respectively), and 10 min after turning to prone position (*prone*_*10*_). For each event 30 s of artefact-free tidal ventilation were extracted. For interventions (suction, adhesive tape begin, adhesive tape end, extubation and NIV) the collected data were divided into 15-s timeframes before and after the event to assess changes occurring during the respective interventions. If an event took place within the 15-s timeframe of a previous event, data were included in both events.

EIT data were extracted and analysed using ibeX (version 1.4, SenTec AG, Landquart, Switzerland). First, vendor-provided predefined anatomical lung regions were projected into the EIT image and EIT signals outside these regions were excluded.^17–19^ Second, EIT signals were extracted and normalized for body weight. Third, the EELI was calculated in arbitrary units per kilogram (AU/kg) for each predefined event. Fourth, changes in EELI were calculated at each event compared to baseline (ΔEELI). Finally, we calculated the tidal volume in AU/kg by measuring EIT signal changes during each inflation provided by the ventilator at baseline (i.e. end-inspiratory minus end-expiratory lung impedance; *V*_T-EIT_). We then correlated these values to the exhaled tidal volume in millilitre per kilogram (ml/kg) measured by the ventilator at the same timepoint to calculate relative changes of FRC (*V*_T_).

### Outcomes

First, we evaluated overall changes in EELI during the entire extubation procedure and then assessed the contribution of each predefined event to changes in EELI. Second, we analysed changes in SpO_2_, FiO_2_ and HR at each of the events and correlated the results to changes in EELI. And finally, we assessed EELI changes within the first ten breaths after initiation of non-invasive ventilation in a breath-by-breath analysis.

### Statistical analysis

Data were analysed using R statistics (version 3.6.1).^20^ Normal distribution was assessed using the Shapiro–Wilks test. Normally distributed data are presented as mean and standard deviation (SD). Non-parametric data are presented as median and interquartile range (IQR). Global changes in EELI, HR, SpO_2_ and SpO_2_/FiO_2_ ratio over all pre-specified events were assessed using a Friedman’s type Skillings–Mack test statistic which tests the global difference in medians over time and provides robust results in case of missing data.^21^ In case of a significant global difference over time, post hoc analyses were performed using paired Wilcoxon tests, corrected for multiple comparisons using the Bonferroni–Holm method. Changes in EELI around an event (e.g. before vs after extubation) were assessed using a paired Wilcoxon test. Correlation was assessed using Spearman’s correlation. Adjusted *p* values < 0.05 were considered statistically significant.

## Results

### Population

Seventeen infants were extubated during the study period and two of the extubations happened accidentally. Three of the remaining 15 extubations occurred during the night where no researcher was available, leaving 12 infants for analysis. Overall, 101 events comprising 2912 breaths were analysed. Patient characteristics are shown in Table 1.

**Table 1:** Baseline demographics and patient characteristics.

Patient characteristics		Median (IQR)
**Perinatal characteristics**		
Gestational age (completed weeks)		27 (27–28)
Birth weight (g)		1140 (951–1152)
Male, *n* (%)		4 (33%)
Complete course of antenatal steroids, *n* (%)		7 (58%)
APGAR score at 5 min		8 (6–8)
**At extubation**		
Postmenstrual age (completed weeks)		28 (27–30)
Age at extubation (days)		3 (2–5)
Weight at extubation (g)^a^		1145 (1068–1250)
Received exogenous surfactant, *n* (%)		12 (100%)
Days of endotracheal ventilation		1 (1–3)
**Ventilator settings before extubation**		
Ventilation mode, *n* (%)		
PSV-VG		10 (83%)
SIMV-VG		2 (17%)
FiO_2_		0.25 (0.21–0.27)
MAP (mbar)		8 (8–10)
*V*_T_ (ml/kg)		4.5 (4.4–5.3)
**Ventilator settings after extubation**		
Ventilation mode, *n* (%)		
NIPPV		5 (42%)
nCPAP		7 (58%)
MAP (mbar)		8 (7–8)

Settings of invasive and non-invasive ventilation were recorded immediately before and immediately after extubation, respectively. *PSV* pressure support ventilation, *SIMV* synchronized intermittent mandatory ventilation, *VG* volume guarantee mode, *FiO*_*2*_ fraction of inspired oxygen, *MAP* mean airway pressure, *V*_*T*_ measured exhaled tidal volume, *NIPPV* nasal intermittent positive pressure ventilation, *nCPAP* nasal continuous positive airway pressure.

^a^For infants ≤72 h, birth weight was used as weight at extubation.

### Timing of extubation procedure

The whole extubation procedure (baseline to prone) took a median (IQR) of 18 (13–23) min and the decisive period from *adhesive tape begin* to *NIV* took 2.3 (1.7–2.8) min (see Supplementary Table S1).

### Changes in lung volumes during the extubation procedure

**Fig. 1:**
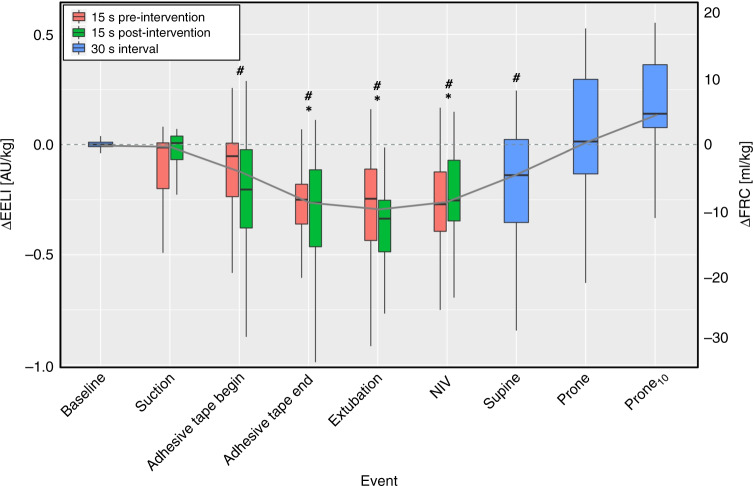
Development of ΔEELI during the extubation process. Red and green boxplots show the 15 s before and after the respective intervention and blue boxplots show results for 30-s intervals without intervention. Overall, ΔEELI changed significantly over time (Friedman’s type Skillings–Mack test: *p* = 0.029). Asterisks and hashtags indicate significant differences in single comparisons of ΔEELI after correction for multiple testing: * = compared to *baseline*; # = compared to *prone*_*10*_. Detailed post hoc comparisons can be found in Supplementary Table S1. ΔEELI = change in end-expiratory lung impedance compared to *baseline*, AU/kg = arbitrary units per kilogram, ΔFRC = change in functional residual capacity compared to baseline, ml/kg = millilitre per kilogram, *NIV =* initiation of non-invasive ventilation after extubation, *prone*_*10*_ = measurement after 10 min in the prone position.

There was a significant change in EELI during the extubation procedure (Friedman’s test, *p* = 0.029), which was attributable to two events: Compared to *baseline*, EELI was significantly lower at *adhesive tape end*, *extubation*, and *NIV* (all *p* < 0.05). At *prone*_*10*_, EELI was significantly higher compared to *adhesive tape begin*, *adhesive tape end*, *extubation*, *NIV* and *supine* (all *p* < 0.05; see Fig. 1, Table 2 and Supplementary Table S2 for detailed results).

**Table 2:** Changes in EELI and FRC compared to baseline (on the left) and compared to the previous event (on the right).

	Changes to baseline			Changes to previous event		
	ΔEELI [AU/kg] median (IQR) difference	ΔFRC [ml/kg] median (IQR) difference	*p*_adj_	ΔEELI [AU/kg] median (IQR) difference	ΔFRC [ml/kg] median (IQR) difference	*p*_adj_
Baseline	–	–	–	–	–	–
Suction	0.0 (−0.18 to 0.02)	0.0 (−6.2 to 0.7)	0.855	0.0 (−0.18 to 0.02)	0.0 (−6.2 to 0.7)	0.855
Adhesive tape begin	−0.09 (−0.33 to 0.0)	−3.1 (−11.3 to 0.0)	0.072	−0.09 (−0.33 to 0.0)	−3.1 (−11.3 to 0.0)	0.072^a^
Adhesive tape end	−0.26 (−0.44 to −0.14)	−8.9 (−15.1 to −4.8)	**0.004**	−0.18 (−0.22 to −0.07)	−6.1 (−2.4 to −7.6)	**0.004**
Extubation	−0.30 (−0.46 to −0.14)	−10.2 (−15.9 to −4.8)	**0.004**	0.05 (−0.01 to 0.16)	1.7 (−0.3 to 5.5)	0.144
NIV	−0.26 (−0.38 to −0.11)	−8.9 (−13.1 to −3.8)	**0.004**	0.02 (−0.04 to 0.08)	0.7 (−1.4 to 2.8)	0.855
Supine	−0.14 (−0.35 to 0.02)	−4.8 (−12.0 to 0.7)	0.06	0.11 (−0.08 to 0.25)	3.8 (−2.8 to 8.6)	0.219
Prone	0.01 (−0.13 to 0.30)	0.3 (4.5 to 10.3)	0.855	0.21 (−0.01 to 0.46)	7.3 (−0.5 to 15.7)	0.106
Prone_10_	0.14 (0.07 to 0.36)	4.8 (2.4 to 12.4)	0.097	0.13 (0.0 to 0.22)	4.5 (0.0 to 7.6)	0.097

*P* values are corrected for multiple testing using the Bonferroni–Holm method. Significant changes are indicated in bold. *ΔEELI* = change in end-expiratory lung impedance compared to baseline, *AU/kg* = arbitrary units per kilogram, ΔFRC = change in functional residual capacity compared to baseline, *ml/kg* = millilitre per kilogram, *NIV* = initiation of non-invasive ventilation after extubation, *prone*_*10*_ = measurement after 10 min in the prone position.

^a^Compared to baseline as not all infants were suctioned endotracheally.

Median (IQR) *V*_T-EIT_ of 0.13 AU/kg (0.10–0.15) corresponded to a *V*_T_ of 4.5 ml/kg (4.4–5.3). At *extubation*, EELI was significantly lower than at *baseline* [median (IQR): −0.30 AU/kg (−0.46 to −0.14), corresponding to an FRC loss of 10.2 ml/kg (4.8–15.9), *p*_adj_ = 0.004]. Loss in EELI between 15 s before and 15 s after extubation was not statistically significant [median difference (IQR): −0.08 AU/kg (−0.15 to 0.0), *p*_adj_ = 0.077]. The biggest decrease in EELI occurred between *adhesive tape begin* and *adhesive tape end* (*p*_adj_ = 0.004) and the biggest increase occurred between *supine* and *prone* (*p*_adj_ = 0.106; see Table 2).

In two infants, EELI did not reach baseline levels at *prone*_*10*_ (see Supplementary Fig. S2): one of them developed a pneumothorax requiring re-intubation 50 h after extubation and the other was relatively mature at 31 completed weeks gestation without further complications.

### Changes in clinical parameters during the extubation procedure

**Fig. 2:**
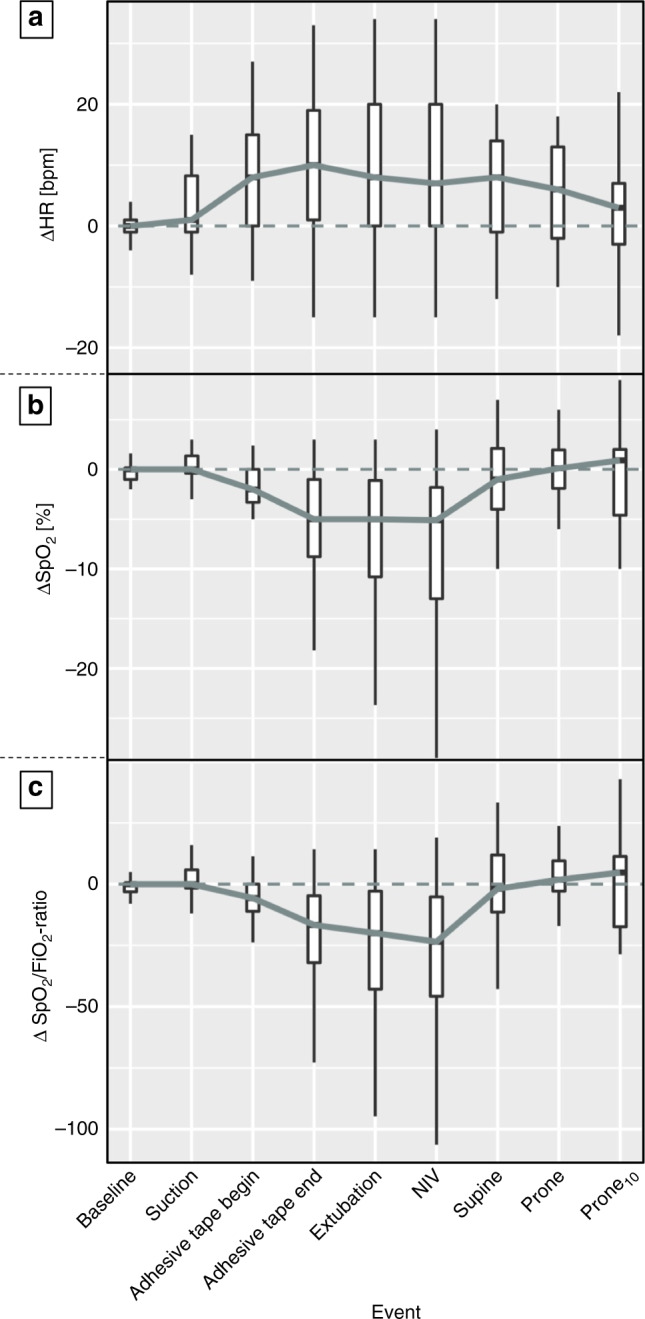
Development of HR (Panel a), SpO_2_ (Panel b), and SpO_2_/FiO_2_ ratio (Panel c) during the extubation procedure. Overall, SpO_2_ changed significantly (*p* = 0.005), while HR (*p* = 0.08) and SpO_2_/FiO_2_ ratio (*p* = 0.12) showed no global changes (assessed using Friedman’s type Skillings–Mack test). *HR =* heart rate, *bpm =* beats per minute, *SpO*_*2*_ = peripheral oxygen saturation, *FiO*_*2*_ = fraction of inspired oxygen, *NIV* = initiation of non-invasive ventilation after extubation, *prone*_*10*_ = measurement after 10 min in the prone position.

Development of HR, SpO_2_ and SpO_2_/FiO_2_ ratio are shown in Fig. 2. There was a significant change in SpO_2_ (Friedman’s test, *p* = 0.006), mainly attributable to a decrease at *adhesive tape end*, *extubation* and *NIV* (see Fig. 2b and Supplementary Table S3). There was a strong correlation of ΔEELI with ΔSpO_2_ (*r* = 0.51, *p* < 0.001) and ΔSpO_2_/FiO_2_ ratio (*r* = 0.48, *p* < 0.001) but not with ΔHR (*r* = −0.18, *p* = 0.10).

**Fig. 3:**
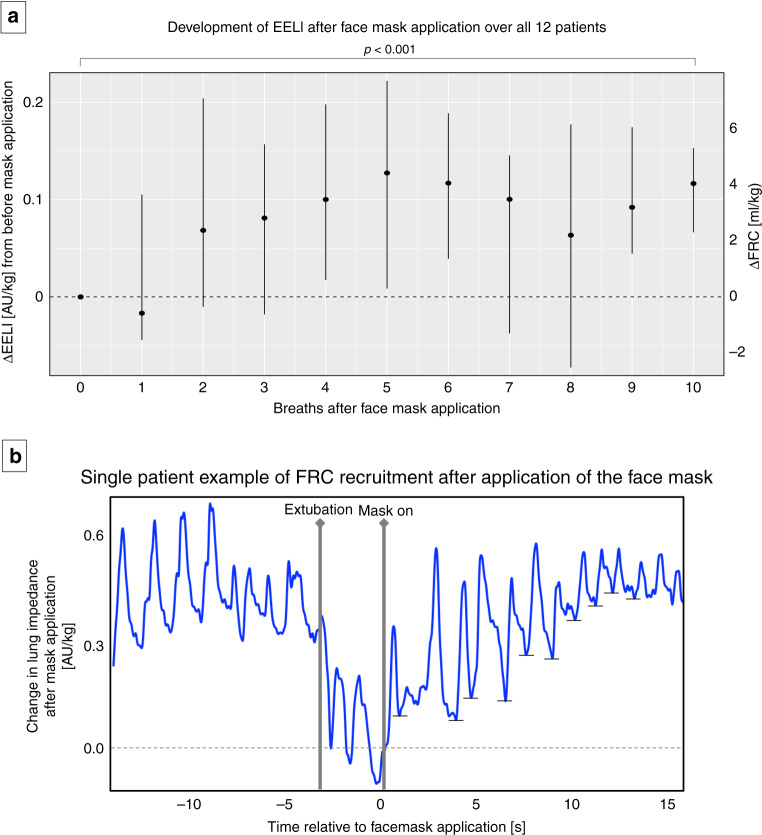
Breath-by-breath analysis of the first ten breaths after initiation of non-invasive ventilation. **a** Changes in EELI over the first ten breaths after initiation of non-invasive ventilation. **b** Changes in lung impedance of an individual patient where the nasal interface was applied quickly after extubation and who responded well to initiation of non-invasive ventilation with a gradual increase in FRC to the level immediately before extubation. Small horizontal lines at the end of each breath indicate the level of end-expiratory lung impedance (EELI).

### Breath-by-breath analysis after initiation of non-invasive ventilation

Thirty-nine per cent of FRC loss during extubation could be regained within the first ten breaths after initiation of non-invasive ventilation [median (IQR) EELI increase at the tenth breath: 0.12 AU/kg (0.07–0.15), corresponding to FRC increase of 4.0 ml/kg (2.3–5.3); *p* < 0.001] (Fig. 3).

## Discussion

This study demonstrated that lung volumes decreased significantly during the extubation procedure of very preterm infants using EIT. Evaluating pre-specified events during the extubation procedure, we showed that adhesive tape removal is the major factor contributing to FRC loss during extubation and that turning the infant prone is helpful in re-establishing FRC. Changes in FRC were highly correlated with changes in SpO_2_ and SpO_2_/FiO_2_ ratio but not with HR. Finally, we could demonstrate that alveolar recruitment started with the first breaths after application of the non-invasive interface.

We described changes in FRC during the complete extubation procedure with lowest values at the time of extubation. We had expected a decrease in FRC but the magnitude of FRC loss (approximately 10 ml/kg) was higher than anticipated. This is twice a regular *V*_T_,^22–24^ and approximately 40% of the FRC of preterm infants at 44 weeks postmenstrual age (21–24 ml/kg measured by multiple breath washout).^25,26^ While the conversion of EIT signal changes to ml/kg has limitations in adults,^27,28^ changes in newborns are representative of the whole lung.^13^ Opposed to other diagnostic tools such as respiratory inductance plethysmography, EIT cannot measure absolute intrapulmonary lung volumes and, therefore, absolute numbers still have to be interpreted cautiously. However, we based our calculations on the ratio of *V*_T_ measurements from the ventilator and from the EIT device which are known to be correlating strongly.^29^ While this approach is limited in its generalizability, our data clearly show a loss in FRC of considerable magnitude which may contribute to physiological instability during and after extubation. This is underlined in the strong correlation of ΔEELI with ΔSpO_2_ and ΔSpO_2_/FiO_2_ ratio in our study.

So far, no study evaluated specific events during the extubation procedure. We now demonstrated that adhesive tape removal was the major factor contributing to FRC loss before extubation, possibly associated with two factors: (1) increased infant movements and concomitant thoracic muscle contractions due to pain which may have resulted in a decreased lung volume; (2) manipulation and movement of the endotracheal tube during adhesive tape removal which may have contributed to increased leak and subsequent FRC loss. Thus, we speculate that re-recruiting atelectatic lung areas before removing the endotracheal tube may be beneficial and propose two potential strategies to achieve re-recruitment: (1) leaving more time between end of adhesive tape removal and extubation and (2) increasing the mean airway pressure (e.g. by 1–2 mbar) after adhesive tape removal but before pulling the endotracheal tube. However, these hypotheses need to be tested in clinical trials before conclusions can be drawn.

After extubation, FRC increased gradually and reached levels higher than during endotracheal ventilation after turning the infant into the prone position. In fact, only two infants did not achieve FRC values as high as prior to extubation, one of which developed a pneumothorax and required re-intubation. Similar to previous reports on the detection of pneumothoraces in preterm infants,^30,31^ this may indicate a benefit of measuring EIT changes during extubation. Prone positioning is associated with an increase in FRC,^14,32^ lung compliance and oxygenation,^33–35^ probably due to a larger area of gas exchange. These factors may have contributed to a higher FRC after extubation. Thus, we speculate that extubating infants in the prone position may improve respiratory stability. However, this hypothesis needs to be tested in further trials.

Finally, re-recruitment of collapsed lung areas after extubation started with the initiation of non-invasive ventilation. Approximately 40% of FRC loss was regained within the first ten breaths after application of a nasal interface. Immediately after birth most breaths of preterm infants are characterized by expiratory braking manoeuvres,^36^ which may assist in establishing and maintaining FRC after birth.^19^ We speculate that similar mechanisms may also have contributed to re-recruitment of FRC after extubation. This finding highlights the importance of immediate initiation of non-invasive ventilation after extubation. We propose two potential means to reduce FRC loss in selected high-risk situations: (1) by pulling back the endotracheal tube only marginally and using it as nasopharyngeal tube temporarily until successful transition to non-invasive ventilation or (2) providing *pre-extubation CPAP* (“*PrePAP*”) by establishing non-invasive ventilation before pulling the endotracheal tube (e.g. via nasal mask or via modified pacifier^37^ depending on route of intubation). However, these ideas need to be tested in adequately powered trials before implementation into clinical practice.

Our study has various limitations: First, we only had a small sample size which may have contributed to the lack of significant changes in HR or SpO_2_/FiO_2_ ratio. However, evaluating approximately 3000 breaths, we could reliably demonstrate changes in EELI during extubation, thereby laying the foundation for subsequent clinical trials. Second, this was an observational study where we chose a pragmatic approach and left all decisions to the treating physicians. This may have resulted in heterogeneity but increased generalizability to clinical practice. Third, we did not focus on clinical outcomes. Based on our data, EIT data may potentially be used as a predictor for extubation failure but this needs to be confirmed in larger studies. Fourth, artefacts induced by body movement or interference with medical devices can affect accuracy of EIT data,^38,39^ but changes in lung volumes over time are known to be accurate in modern devices.^12^ Our findings are useful for the development of future trials which may ultimately reduce re-intubation and mechanical ventilation, thereby potentially reducing relevant long-term morbidity and mortality in preterm infants.^4,40,41^ Fourth, this was a single-centre study limiting the generalizability of our findings, particularly as extubation procedures vary between neonatal units.

## Conclusion

In this study, we demonstrated that lung volumes decreased significantly during the extubation procedure of very preterm infants. Removal of adhesive tape is the major factor contributing to FRC loss during extubation and turning the infant prone after extubation is helpful in re-establishing FRC. Changes in FRC were highly correlated with changes in SpO_2_ and SpO_2_/FiO_2_ ratio. Finally, re-recruitment of FRC starts with the first breaths after initiation of non-invasive respiratory support. This observational study provides relevant information for designing new trials investigating the optimal way of extubating a preterm infant.

## Supplementary information

Supplementary checklist

Supplementary Material

## Data Availability

De-identified individual participant data, study protocols and statistical analysis codes are available from 3 months to 10 years following article publication to researchers who provide a methodologically sound proposal, with approval by an independent review committee (“learned intermediary”). Proposals should be directed to vincent.gaertner@usz.ch to gain access. Data requestors will need to sign a data access or material transfer agreement approved by USZ.
