# Supplementary material for: The DELUX study: development of lung volumes during extubation of preterm infants
Source: Pediatr Res. 2021 Aug 31;92(1):242–8. doi: 10.1038/s41390-021-01699-w (PMC8406659; doi:10.1038/s41390-021-01699-w)
Supplement: Supplementary file 2 — Supplementary Material [file 41390_2021_1699_MOESM2_ESM.docx]

**The DELUX study – Development of Lung volumes during extubation of preterm infants**

Leonie Plastina^1^†, Vincent D. Gaertner^1^†*, Andreas D. Waldmann^2^, Janine Thomann^1^, Dirk Bassler^1^, Christoph M. Rüegger^1^

^1^ Newborn Research, Department of Neonatology, University Hospital and University of Zurich, Zurich, Switzerland

^2^ Department of Anesthesiology and Intensive Care Medicine, Rostock University Medical
 Center, Rostock, Germany

† These authors contributed equally.

* Corresponding author

**SUPPLEMENTARY MATERIAL**

**Supplementary Tables**

| **Time interval** | **Median (IQR) (seconds)** |
| --- | --- |
| Baseline – suction | 45 (35 – 85) |
| Suction – plaster begin | 308 (300 – 788) |
| Adhesive tape begin – adhesive tape end | 75 (70 – 193) |
| Adhesive tape end – extubation | 19 (16 – 27) |
| Extubation – NIV | 19 (14 – 22) |
| NIV – supine | 496 (306 – 788) |
| Supine – prone | 88 (52 – 129) |
| Prone – prone10 | 595 (575 – 600) |

**Supplementary Table S1. Time intervals during the extubation procedure in seconds.** *Abbreviations*: NIV = initiation of non-invasive ventilation after extubation, prone_10_ = measurement after 10 minutes in prone position.

|  | Baseline | Suction | Adhesive tape begin | Adhesive tape  end | Extubate | NIV | Supine | Prone | Prone_10_ |
| --- | --- | --- | --- | --- | --- | --- | --- | --- | --- |
| Baseline | NA |  |  |  |  |  |  |  |  |
| Suction | 0.855 | NA |  |  |  |  |  |  |  |
| Adhesive tape begin | 0.072 | 0.508 | NA |  |  |  |  |  |  |
| Adhesive tape end | **0.004** | 0.105 | **0.004** | NA |  |  |  |  |  |
| Extubate | **0.004** | 0.161 | 0.054 | 0.144 | NA |  |  |  |  |
| NIV | **0.004** | 0.161 | 0.085 | 0.116 | 0.855 | NA |  |  |  |
| Supine | 0.06 | 0.161 | 1 | **0.03** | 0.105 | 0.219 | NA |  |  |
| Prone | 0.855 | 0.703 | 0.059 | **0.004** | **0.008** | **0.018** | 0.106 | NA |  |
| Prone_10_ | 0.097 | 0.3 | **0.038** | **0.004** | **0.004** | **0.004** | **0.038** | 0.097 | NA |

**Supplementary Table S2. P-values of post-hoc analyses for development of ΔEELI during extubation.** Results for paired Wilcoxon tests with Bonferroni-Holm correction. Significant differences are printed bold. *Abbreviations*: NIV = initiation of non-invasive ventilation after extubation, prone_10_ = measurement after 10 minutes in prone position.

|  | Baseline | Suction | Adhesive tape begin | Adhesive tape  end | Extubate | NIV | Supine | Prone | Prone_10_ |
| --- | --- | --- | --- | --- | --- | --- | --- | --- | --- |
| Baseline | NA |  |  |  |  |  |  |  |  |
| Suction | 1 | NA |  |  |  |  |  |  |  |
| Adhesive tape begin | 0.13 | 0.18 | NA |  |  |  |  |  |  |
| Adhesive tape end | **0.036** | 0.21 | 0.24 | NA |  |  |  |  |  |
| Extubate | **0.036** | 0.18 | 0.1 | 0.72 | NA |  |  |  |  |
| NIV | **0.030** | 0.18 | 0.05 | 0.14 | **0.039** | NA |  |  |  |
| Supine | 0.88 | 0.78 | 0.30 | **0.030** | **0.030** | **0.030** | NA |  |  |
| Prone | 0.87 | 0.96 | 0.21 | **0.032** | **0.030** | **0.030** | 0.99 | NA |  |
| Prone_10_ | 0.99 | 0.75 | 0.34 | 0.05 | **0.030** | **0.030** | 0.62 | 0.9 | NA |

**Supplementary Table S3. P-values of post-hoc analyses for development of SpO2 during extubation.** Results for paired Wilcoxon tests with Bonferroni-Holm correction. Significant differences are printed bold. *Abbreviations*: NIV = initiation of non-invasive ventilation after extubation, prone_10_ = measurement after 10 minutes in prone position.

**Supplementary Figures**

**
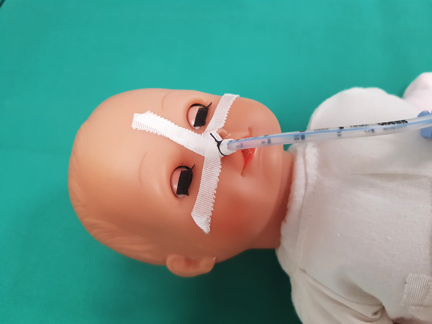
**

**Supplementary Figure S1. Demonstration of nasal endotracheal tube fixation.**

**
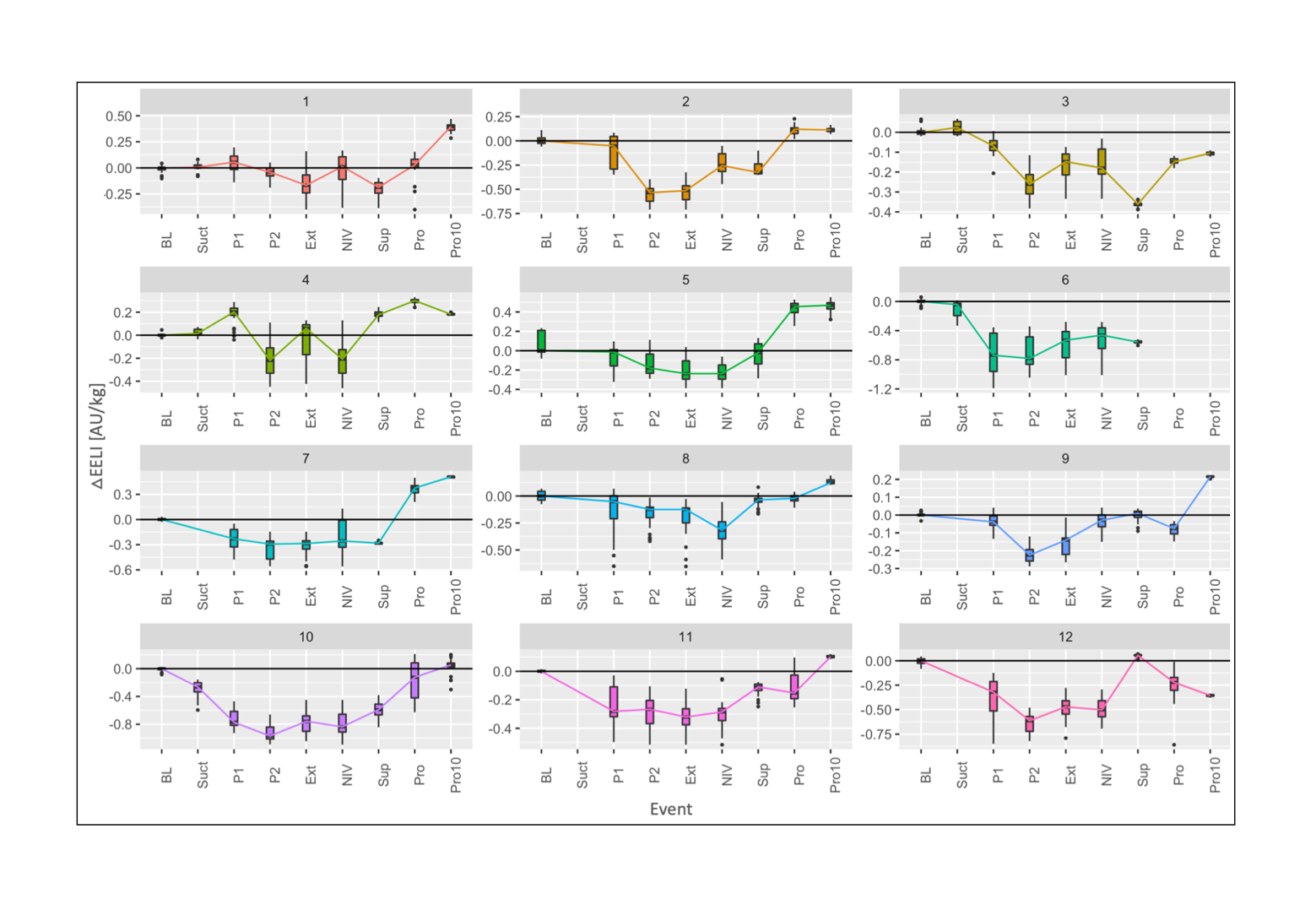
**

**Supplementary Figure S2. Development of EELI over time for all patients.** Black lines in each graph demonstrate baseline EELI values. Of note, in patient #6 there was a loss of contact from the EIT electrodes while turning the infant into prone position resulting in loss of evaluable data. *Abbreviations*: BL = baseline, Suct = endotracheal suctioning, P1 = begin adhesive tape removal, P2 = end of adhesive tape removal, Ext = extubation, NIV = initiation of non-invasive ventilation, Sup = supine, Pro = prone, Pro10 = 10 minutes after turning to prone position.
